# Construction of Electrochemical and Photoelectrochemical Sensing Platform Based on Porphyrinic Metal-Organic Frameworks for Determination of Ascorbic Acid

**DOI:** 10.3390/nano12030482

**Published:** 2022-01-29

**Authors:** Xin Xu, Chuan-Hua Li, Hong Zhang, Xi-Ming Guo

**Affiliations:** Harbin Institute of Technology, School of Life Science of Technology Institution, 92 West Dazhi Street, Nan Gang District, Harbin 150006, China; 55449344@qq.com (X.X.); 1989833301@qq.com (C.-H.L.); 2298439924@qq.com (H.Z.)

**Keywords:** Cu-TCPP@MOFs, ascorbic acid, cyclic coltammetry (CV), photoelectrochemical, differential pulse voltammetry (DPV), chronoamperometry (CA)

## Abstract

Highly sensitive and specific detection of biomolecular markers is of great importance to the diagnosis and treatment of related diseases. Herein, Cu-TCPP@MOFs thin films were synthesized with tetrakis(4-carboxyphenyl) porphyrin (H_2_TCPP) as organic ligands and copper ions as metal nodes. The as-synthesized Cu-TCPP@MOFs thin films as electrode modifiers were used to modify the pre-treated glassy carbon electrode (GCE) and the electrochemical performances of Cu-TCPP@MOFs/GCE were evaluated by electrochemical impedance spectroscopy (EIS) and cyclic voltammetry (CV). Furthermore, as the working electrode, the constructed Cu-TCPP@MOFs/GCE was used for the investigation of ascorbic acid (AA) due to its outstanding electrocatalytic activities towards AA by several electrochemical methods, including cyclic voltammetry (CV), differential pulse voltammetry (DPV), and chronoamperometry (CA). The well-linear relationship was established based on different AA concentration ranges and the ideal detection limits (LOD) were obtained in the above-mentioned electrochemical methods, respectively. Furthermore, a Cu-TCPP MOFs@GCE sensing platform was used as a photoelectrochemical (PEC) sensor to quantitatively detect AA based on the strong absorption properties of Cu-TCPP ingredients in Cu-TCPP MOFs in a visible light band of 400~700 nm. PEC sensing platform based on Cu-TCPP@MOFs exhibited a more extensive linear concentration range, more ideal detection limit, and better sensitivity relative than the other electrochemical methods for AA. The well linear regression equations were established between the peak current intensity and AA concentrations in different electrochemical technologies, including CV, DPV, and CA, and PEC technology. AA concentration ranges applicable to various electrochemical equations were as follows: 0.45~2.10 mM of CV, 0.75~2.025 mM of DPV, 0.3~2.4 mM of CA, 7.5~480 μM of PEC, and the corresponding detection limits for AA were 1.08 μM (S/N = 3), 0.14 μM (S/N = 3), 0.049 μM (S/N = 3), and 0.084 nA/μM. Moreover, the proposed Cu-TCPP MOFs@GCE electrochemical and photoelectrochemical sensing platform was applied to determine the AA concentration of a real human serum sample; the results reveal that Cu-TCPP MOFs@GCE sensing platform could accurately determine the concentration of AA of the human serum under other potential interferences contained in the human serum samples.

## 1. Introduction 

Ascorbic acid (AA), as a necessary vitamin, cannot be self-synthesized by human tissue due to a genetic mutation that causes a deficiency in L-gulonol lactone oxidase. Generally, the human body needs AA only ingested through exogenous food, beverage, and pharmaceutical formulations [1]. Normal AA levels could perform essential physiological behaviors in many biological activities of various biological species. However, abnormal intake of AA is harmful to human health. (i) In food and beverages, excessive intake of AA could lead to side effects such as gastric irritation, diarrhea, exanthema, skeletal diseases, polyuria, oxalic acid, and uric acid stones [2], while the lack of AA could result in cardiovascular diseases. (ii) In pharmaceutical formulations, AA as a strong active oxygen scavenger could protect the brain against oxidative stress reactions. Meanwhile, AA as a neuromodulator participates in various neuronal information transmission and regulation of electrical activities [3]. Therefore, insufficient AA results in ischemia, Alzheimer’s disease, and Parkinson’s disease [4]. Inversely, excessive AA leads to the same side effects in food and beverages. Hence, the concentration level of AA in various organisms could be used as a potential preventive and therapeutic indicator of various associated diseases caused by abnormal levels of AA. 

To meet the requirements of the quantitative detection of AA in clinical timely examination and practical production of food and beverages, the construction of the simple, well-suited, rapid, sensitive, and accurate AA detection method is crucial. Up to date, various novel and advanced analysis methods have been explored to the quantitative determination of AA, mainly including colorimetric [5], fluorescence methods [6], high-performance liquid chromatography (HPLC) [7], high-performance thin-layer chromatography (HPTLC) [8], liquid chromatography-mass spectrometry (LC-MS) [9], capillary electrophoresis [10], fluorescence spectrophotometry [11], and chemiluminescence spectroscopy [12]. However, these analysis methods have many insurmountable limitations, including samples requiring pre-treatment, operation steps, complicated analysis procedures, time-consuming, expensive instruments, and the additional use of chemical reagents. Whereupon, scientists are continually searching for a better method to detect AA in different human components [13]. Electrochemical technology as a feasible analysis method has been gradually approved because many characteristics including its specificity, rapid response, high sensitivity, simple operation, and low expense were superior to that of the above-mentioned other methods [14,15,16]. In addition, the targeted molecule was simultaneously detected by the collaborative application of various electrochemical inspection technology, which has better selectivity and flexibility for the same detection target in different environments. The selectivity, sensitivity, and detection limit of the target biomolecule are strictly determined by the characteristics of electrode modifiers. Therefore, many researchers have devoted their efforts to designing and preparing various electrode modifiers, including, but not limited to nanoparticles [17], nanocomposite [18], metal-organic frameworks (MOFs) [19,20,21], carbon nanotubes [22], graphene, graphene oxide [23], coordination polymer [24], ionic liquid [4] and so on. Among these electrode modifiers, MOFs were used as excellent electrode modifiers due to their facile and environmentally friendly synthesis approaches, unique topological structure features and functions including permanently porous structure, uniform channels, large internal surface areas, thermal stability, nanometer-sized cavities, and excellent catalytic activity. First, the porous structure and large specific surface area of MOFs provide more interfaces and active sites for the interactions between analyst and material. Secondly, the organic ligands with rich functional groups make MOFs easily functionalized with various molecules and other materials. Third, the diverse metal and organic ligands of MOFs offer excellent catalytic activities [25]. The topological structural features and functions of MOFs could be well-regulated by the selection of different coordination metal ions or clusters, including transition metal elements, parts of group elements, rare earth metals and organic ligands, including oxygen-containing ligands (carboxylic acid ligands, etc.), nitrogen-containing ligands (nitrogen heterocyclic ligands, etc.) and both nitrogen and oxygen atoms (nitrogen-heterocyclic carboxylic acid ligands, etc.) [26,27]. The transition metal element copper with a highly active intermediate valence state and suitable coordination space (generally 4~6 coordination bonds) is an appropriate block metal node. Cu centers may be involved in electrochemical reduction-oxidation processes because Cu^2+^ can “shuttle” between transient reduced states such as Cu^+^ and Cu^0^ or transient oxidation states such as Cu^3+^ and even Cu^4+^ [28]. H_2_TCPP acted as an appropriate organic linker due to their excellent characteristics, such as excellent interfacial self-assembly characteristics, outstanding molecular recognition through axial coordination effect, perfect visible light-harvesting capability, strong electrocatalytic and photocatalytic activity [29,30]. Therefore, the copper ions and H_2_TCPP were selected as fundamental building units to construct Cu-TCPP@MOFs. Cu^2+^ is easily coordinated with four pyrrole nitrogen of H_2_TCPP and formed Cu-TCPP metalloporphyrin because the radius of Cu^2+^ (the ionic radius is 72 pm) was well-matched with the tetrapyrrole structure scale of H_2_TCPP in Cu-TCPP@MOFs [31]. The formation of Cu-TCPP could effectively improve the stability of Cu-TCPP@MOFs. Meanwhile, Cu-TCPP@MOFs have superior physical, chemical, optical characteristics, and excellent catalytic activities. Cu-TCPP@MOFs were used as electrode modification materials to construct electrochemical sensors with the following advantages. Firstly, Cu-TCPP@MOFs with superior surface area and high porosity could increase the effective contact between Cu-TCPP@MOFs and the targeted molecule, which could lead to signal amplification for the targeted molecule in electrochemical sensing procedures. Secondly, Cu-TCPP@MOFs has strong absorption in visible light due to the highly conjugated degree and delocalized π bonds of Cu-TCPP ligand, which could be constructed not only by electrochemical sensors but also photoelectrochemical (PEC) sensors in the visible region. Furthermore, thanks to the thin films with larger specific surface areas, more exposed active centers, and smaller diffusion resistance, Cu-TCPP@MOFs thin films had superior electrochemical and PEC activities relative to that of bulk materials [32].

In the manuscript, Cu-TCPP@MOFs thin films were successfully prepared by a simple solvothermal route and employed as an electrochemical and PEC biosensor for quantitative detection of AA. The electrochemical performances of Cu-TCPP@MOFs/GCE were evaluated by EIS and CV. The results of EIS showed that R_ct_ of Cu-TCPP@MOFs/GCE reduced 97.58% compared with that of bake GCE, therefore, we explored AA electrochemical and PEC sensing platforms based on Cu-TCPP@MOFs thin films. 

## 2. Experimental

### 2.1. Materials

All chemical reagents were purchased from Shanghai Chemical Reagent Company (Shanghai Chemical Reagent Company, Shanghai, China). All the reagents were of analytical reagent grade and without further purification before use. Nafion was brought from Sigma (Sigma Aldrich Co., Saint Louis, MO, USA). Phosphate buffer solution (PBS) was composed of 0.1 M Na_2_HPO_4_ and 0.1 M NaH_2_PO_4_. The pH was adjusted by 0.1 M HCl and measured by pH meter PB-10 (Sartorius Co., Gottingen, Germany). AA in PBS (0.1 M pH 7.0) were prepared before use. All aqueous solutions were prepared using deionized water.

### 2.2. Synthesis of Cu-TCPP@MOFs and Preparation of the Modified Electrodes 

The synthetic procedure and characterization of Cu-TCPP@MOFs have been previously described by the authors elsewhere [33]. The synthesis procedure of Cu-TCPP@MOFs was briefly described as follows: Cu-TCPP@MOFs were synthesized by using a mixture of J-aggregate H_2_TCPP (20 mg, 0.025 mmol) and Cu (NO_3_)_2_·3H_2_O (100 mg, 0.139 mmol) in 18 mL of mixed solvent (DMF: ethanol = 1:1). Then, the solution was stirred for 10 min and transferred into a 25 mL Teflon-lined autoclave, sealed and maintained at 85 ℃ for 48 h. The synthesized powder was collected and washed successively with DMF, water, and ethanol. Then, Cu-TCPP@MOFs were obtained after being dried at room temperature.

For the working electrode preparation, the polished GCE with 0.03 and 0.05 mm alumina slurry was ultrasonically washed with doubly distilled water and ethanol, respectively. Then, 1 mg Cu-TCPP@MOFs were ultrasonically dispersed into 1 mL DMF including 0.2% Nafion to obtain 1 mg/mL well-dispersed Cu-TCPP@MOFs DMF suspension. Then, 5 μL of the suspension was drop-coated onto the surface of pre-treated bare GCE and exposed to air for 12 h at room temperature (23 ℃ ± 2 ℃) before electrochemical measurements.

### 2.3. Characterization and Electrochemical Measurements

UV-Vis absorption spectrum was performed using a U-3900 UV-Vis spectrometer (Hitachi Co., Tokyo, Japan). Scanning electron microscopy (SEM) was performed with a Quanta 200 scanning electron microscope (FEI Co., Eindhoven, The Netherlands), and X-ray diffraction (XRD) patterns were recorded on an X’PERT Malvern Panalytical X-ray diffractometer with Cu Kα radiation (λ = 1.5406 Å) (Panalytica Co., Almelo, The Netherlands). The chemical compositions of the as-prepared sample was measured by using energy dispersive X-ray spectroscopy (EDS) (FEI Co., The Netherlands).

The electrochemical measurements were performed using a Bio-Logic VSP-300-6 electrochemical working station (Bio-Logic Company, Seyssinet-Pariset, France). The electrochemical experiment was conducted on a three-electrode system, including Cu-TCPP@MOFs/GCE as a working electrode, a platinum wire (φ = 3 mm) as an auxiliary electrode and Ag/AgCl/KCl (saturated potassium chloride solution) as a reference electrode, and PBS (0.1 M pH = 7.0) as the supporting electrolyte. All potentials were quoted versus saturated calomel electrode (SCE) or Ag/AgCl/KCl (saturated potassium chloride solution) reference electrode. CV scans were performed from −0.6 V to 0.6 V, and potential cycling was carried out between −0.6 V and 0.6 V.

## 3. Results and Discussion

### 3.1. Characterization of the Samples

#### 3.1.1. XRD of Cu-TCPP@MOFs 

**Figure 1:**
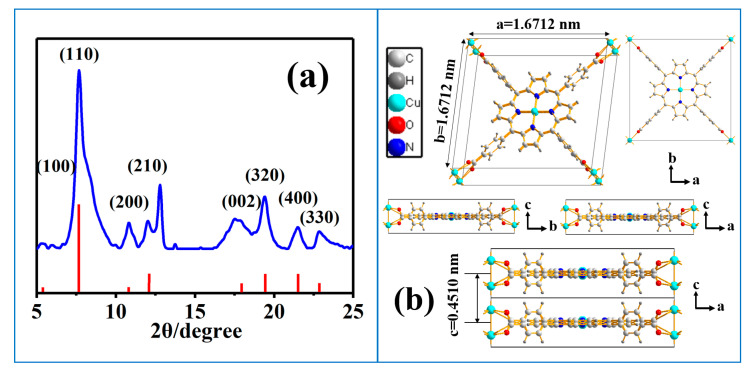
(**a**) XRD pattern of Cu-TCPP@MOFs, smoothed with Jade 6.0. (**b**) Projections of the proposed crystal structure of Cu-TCPP@MOFs on the ab plane, bc plane, and ac plane.

XRD analysis was conducted to investigate the crystal structure of as-prepared Cu-TCPP@MOFs. The XRD patterns of Cu-TCPP@MOFs were recorded in Figure 1a. The distinguishable XRD diffraction peaks observed at 5.44°, 7.96°, 11.02°, 11.97°, 18.12°, 19.40°, 21.48°, and 22.85° were indexed to Cu-TCPP@MOFs planes of (100), (110), (200), (210), (002), (320), (400) and (330) according to the previous structure reported by us [34]. The lattice parameters are in agreement with a “checkerboard” structure consisting of Cu-TCPP connected by paddle-wheel binuclear Cu_2_(COO)_4_ along the ab plane and the layers are stacked along the C axis without interleaving and overlapping. Along the c axis, two “checkerboard” layers are vertically wrapped within the lattice (Figure 1b). According to Bragg’s law, the value of c in the crystal lattice is 0.4510 nm, which is double vertical distance between two neighboring porphyrin layers. 

#### 3.1.2. SEM and EDS of Cu-TCPP@MOFs

**Figure 2:**
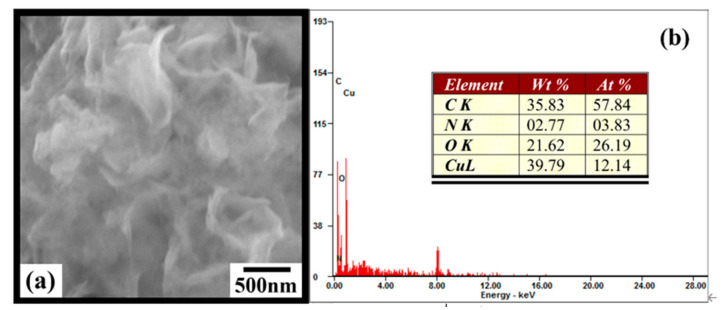
(**a**) SEM image of Cu-TCPP@MOFs. (**b**) EDS analysis of Cu-TCPP@MOFs.

The morphological characteristics of Cu-TCPP@MOFs were investigated by SEM, and the results are shown in Figure 2a. SEM image showed the morphology of Cu-TCPP@MOFs were thin films with spontaneous crimp stacking tendency. The crystal structure model can also well support the formation of the morphology of Cu-TCPP@MOFs thin films. The coordination bonds along the ab plane between the Cu atom and the carboxyl group are much stronger than the hydrogen-bonding and van der Waals among the checkerboard layers along the c axis, which would induce crystal growth along the ab plane faster than that along the c axis during crystallization. EDS were employed for compositional analysis of as-prepared Cu-TCPP@MOFs and is displayed in Figure 2b. The Cu/N atomic ratio is important for determining the composition of Cu-TCPP@MOFs, since nitrogen content in the organic linkers is determined, and the nitrogen does not participate in the construction of a basic MOFs topological unit. The measured Cu/N atomic ratio is 3.12, which is close to the simulated value of Cu-TCPP@MOFs with spherical morphology in our previous report [34]. The synthesis methods of Cu-TCPP@MOFs with spherical morphology and thin films morphology are very similar, and the types and concentrations of organic ligands and metal salts used are the same, so although the morphologies are different, the element ratios are similar.

### 3.2. Characterization of the Modified Electrode 

**Figure 3:**
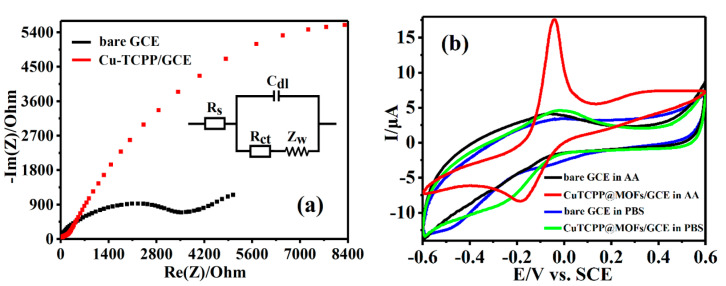
(**a**) EIS of 10 mM [Fe(CN)_6_]^3−^/^4−^ containing 0.1 M KCl using different materials modified GCEs. (**b**) CVs of 0.1 M PBS (pH = 7.0) including 1.875 mM AA at different electrodes, and CV of 0.1 M PBS (pH = 7.0) at different electrodes within the electrochemical window from −0.6 V to 0.6 V. Scan rate: 90 mV·s^−1^.

To further understand the electrochemical performances of Cu-TCPP@MOFs, EIS was used to analyze the conductivity of bare GCE and Cu-TCPP@MOFs/GCE in 10 mM [Fe(CN)_6_]^3−/4−^ containing 0.1 M KCl in the frequency range from 100 kHz to 0.1 Hz (Figure 3a). The equivalent circuit of electrolyte consists of resistance (Rs), double layer capacitance (C_dl_), and Faraday impedance (Z_f_). Z_f_ usually consists of serially connected charge transfer resistance (R_ct_) and Warburg impedance (Z_w_). R_ct_ value obtained from the diameter of the semicircle in the Nyquist plot was applied to describe the conductivity and electron-transfer properties of the electrodes. The lower R_ct_ value could indicate the better conductivity and faster electron transfer rate of the measured electrode [35]. The R_ct_ value 3198.20 Ω of GCE was much higher than the R_ct_ value 77.53 Ω of Cu-TCPP@MOFs/GCE, which illustrated excellent electron communication between Cu-TCPP@MOFs and GCE. In Cu-TCPP@MOFs, H_2_TCPP ligand and copper ion coordinated and formed Cu-TCPP, which was linked with four Cu paddlewheel metal nodes to form a layered Cu-TCPP@MOFs thin film [36]. The strong π-stacked conjugation and the heteroatom of Cu-TCPP could enhance electron transport to both Cu-TCPP@MOFs and GCE. More specifically, many Cu-O-C groups as electron transport mediums greatly accelerated the electron communication between GCE and Cu-TCPP@MOFs, which could provide a good basis for electrochemical sensors.

CV was an effective method to study the electrocatalytic activity of modified electrode for analysts. CV curves of bare GCE and Cu-TCPP@MOFs/GCE were measured in 0.1 M PBS (pH = 7.0) and PBS (0.1 M, pH 7.0) including 1.875 mM AA at 90 mV·s^−1^ within the electrochemical window from −0.6 V to 0.6 V, respectively, as shown in Figure 3b. The bare GCE and Cu-TCPP@MOFs/GCE showed no obvious electrochemical response current peak in PBS solution, which indicates that there is no corresponding electrode reaction. Furthermore, the bare GCE displayed an ill-defined electrochemical response current peak because of the lethargic electrochemical response in 0.1 M PBS (pH = 7.0) including 1.875 mM AA, which was similar to previous reports [37]. Cu-TCPP@MOFs/GCE promoted electrochemical response of AA compared to bare GCE. An obvious oxidation-reduction appears at about −42 mV and −186 mV at Cu-TCPP@MOFs/GCE, which shows efficient catalytic activities of Cu-TCPP@MOFs/GCE toward AA. On the other hand, the oxidation peak current intensity is twice higher than that of the reduction peak, which could be attributed to the fact that the oxidation process is a two electron process and the reduction process was a single electron process. 

### 3.3. Effect of Scan Rate on the Peak Currents of AA

**Figure 4:**
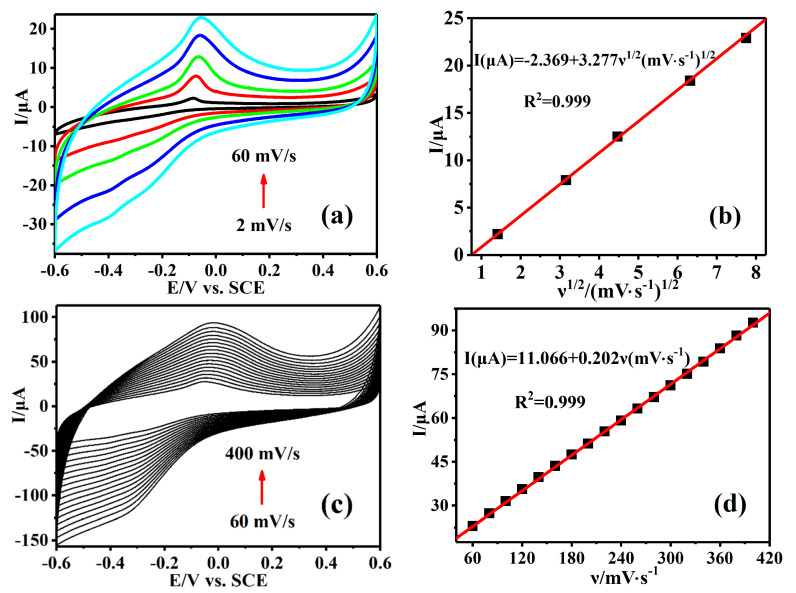
(**a**) CVs of Cu-TCPP@MOFs/GCE in 1.25 mM AA in PBS (0.1 M, pH = 7.0) under relatively low scan rate (2, 10, 20, 40 and 60 mV·s^−1^). (**b**) The standard curve of the peak currents versus different scan rate (2~60 mV·s^−1^). (**c**) CVs of Cu-TCPP@MOFs/GCE in 1.25 mM AA in PBS (0.1 M, pH = 7.0) under relatively high scan rate (60, 80, 100, 120, 140, 160, 180, 200, 220, 240, 260, 280, 300, 320, 340, 360, 380 and 400 mV·s^−1^). (**d**) The standard curve of the peak currents versus different scan rate (60~400 mV·s^−1^).

The effect of scan rate on CV curves of AA was studied with Cu-TCPP@MOFs/GCE as a working electrode in AA solution. The changes of the peak current intensity were regarded as the different electrode reaction properties with the increase in scanning rate. Figure 4a shows the electrochemical behavior of Cu-TCPP@MOFs/GCE at scanning rates of 2~60 mV·s^−1^ and the peak current intensity is proportional to the square root of scan speed, indicating that the electrode reaction of AA on Cu-TCPP@MOFs/GCE surface is a diffusion control process in the lower scanning rates (Figure 4b). Figure 4c shows the electrochemical behavior of Cu-TCPP@MOFs/GCE at scanning rates of 60~400 mV·s^−1^ and the peak current is proportional to the scanning rates, indicating that the electrode reaction of AA on Cu-TCPP@MOFs/GCE surface is a typical surface adsorption control process (Figure 4d). In general, diffusion control and adsorption control exist simultaneously in the electrode surface reaction process, and the electrochemical reaction and electrolyte diffusion are mutually restricted. On Cu-TCPP@MOFs/GCE, at a relatively small scanning rate (2~60 mV·s^−1^), diffusion control is dominant. At the moment, AA has a relatively sufficient time to adsorb on Cu-TCPP@MOFs with a large specific surface area, and the diffusion velocity becomes the controllable factor restricting the reaction. On the contrary, while at a relatively large scanning rate (60~400 mV·s^−1^), adsorption control is dominant, which might be attributed to the fact that the diffusion rate of AA in solution from the substrate to Cu-TCPP@MOFs/GCE was not fast enough to satisfy the speed of consumption. 

### 3.4. CV of AA on Cu-TCPP@MOFs/GCE

**Figure 5:**
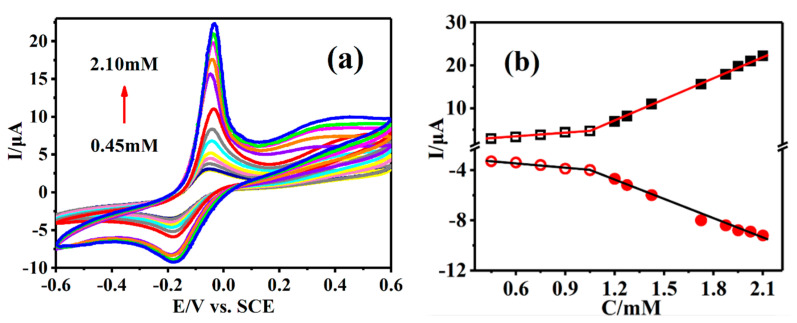
(**a**) CVs of Cu-TCPP@MOFs/GCE in different concentrations of AA solution. (**b**) The standard curves of the peak currents versus different concentrations of AA solution.

The sensing platform based on Cu-TCPP@MOFs/GCE was used to detect the target molecule AA under the different AA concentration solutions in the carrier electrolyte PBS (pH = 7.0), by CV electrochemical technology under the specific potential window. Figure 5a shows that a couple of oxidation and reduction peaks are obviously observed at Epc = −42 mV and Epa = −186 mV, which was assigned to the catalytic oxidation and reduction current peak on Cu-TCPP@MOFs/GCE, respectively. The oxidation and reduction current response intensity for AA on Cu-TCPP@MOFs/GCE is concentration dependent. The oxidation peak current intensity at −42 mV enhances gradually with the increase in AA concentration and an ideal linear relationship is established between the peak current intensity and different AA concentrations. The linear equation corresponding to AA concentration range from 0.45 to 1.05 mM is Ipc(µA) = 1.470 + 3.133C(mM), R^2^ = 0.991 and AA concentration varied from 1.05 to 2.10 mM, the corresponding line relationship is Ipc(µA) = −13.051 + 16.735C(mM) R^2^ = 0.999. Similarly, the changing trend of reduction peak current intensity at −186 mV was the same as that of the above oxidation peak current on Cu-TCPP@MOFs/GCE, and also enhanced gradually with the increase in AA concentration. Additionally, the corresponding linear equations of AA concentration intervals are also established. Their linear equations are Ipa(µA) = −2.690−1.267C(mM), R^2^ = 0.970 in AA concentration ranges from 0.45 to 1.05 mM and Ipa(µA) = 1.346−5.149C(mM), R^2^ = 0.989 in AA concentration ranges from 1.05 to 2.10 mM, respectively (Figure 5b). 

### 3.5. DPV of AA at Cu-TCPP@MOFs/GCE

**Figure 6:**
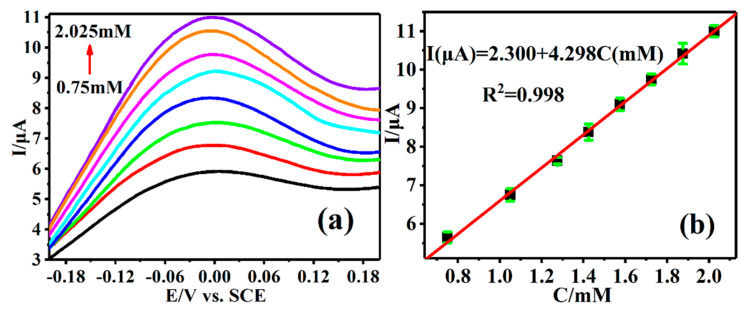
(**a**) DPVs of Cu-TCPP@MOFs/GCE in different concentrations of AA solution. (**b**) The standard curves of the peak currents versus different concentrations of AA solution.

As the sensitivity of DPV technology is higher than that of CV, DPV technology was used to detect the different AA concentrations by the Cu-TCPP@MOFs/GCE sensing platform. Figure 6a shows that the peak current response on Cu-TCPP@MOFs/GCE towards AA appeared at −0.42 mV, which is identical to the oxidation peak in CV technology, therefore, the current is attributed to the oxidation peak of AA on Cu-TCPP@MOFs/GCE. The current intensity of the oxidation peak of AA is concentration-dependent and the enhancement of current intensity with the increase in AA concentration in DPV technology and an ideal linear relationship between the peak current and AA concentration is established. The corresponding linear regression equation is I(µA) = 2.300 + 4.298*C*(mM) R^2^ = 0.998 in AA, concentration ranges are from 0.75 to 2.025 mM (Figure 6b), the corresponding detection sensitivity is 4.298 µA/mM, and the corresponding lowest detection limitation is 0.14 µM.

### 3.6. Amperometric I-t Response of AA at Cu-TCPP@MOFs/GCE

**Figure 7:**
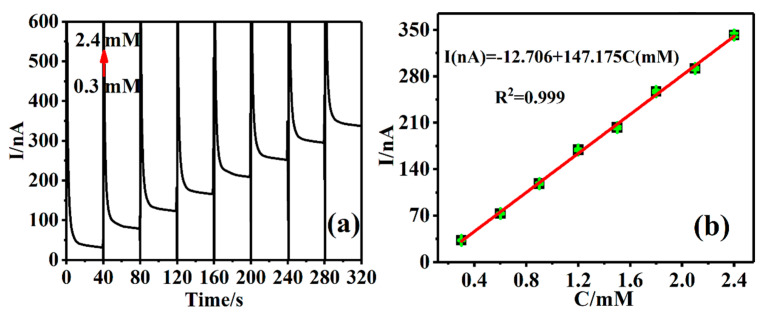
(**a**) Amperometric I-t response at Cu-TCPP@MOFs/GCE in 0.1 M PBS (pH = 7) applied potential of 0.1 V; (**b**) The standard curves of the peak currents versus different concentrations of AA solution.

In order to further expand the application concentration range of the Cu-TCPP@MOFs/GCE sensing platform for AA detection, the Amperometric I-t method was used to detect different AA concentrations on the Cu-TCPP@MOFs/GCE sensing platform. The measured results are shown in Figure 7a: the recorded signals of Cu-TCPP@MOFs/GCE sensing platform increased with the increase in AA concentrations from 0.3 mM to 2.4 mM. The corresponding linear regression equation is I(nA) = −12.706 + 147.175*C*(mM), R^2^ = 0.999 (Figure 7b), the lowest detection limit is 0.049 µM and the sensitivity is 0.147 nA/mM.

### 3.7. “On-Off-On” PEC Sensing Platform of Cu-TCPP@MOFs/GCE

A PEC sensing platform is a target analyzing device based on photoelectric active material. The PEC sensing platform has attracted more and more attention due to its advantages such as fast response, good portability, real-time detection, high sensitivity, well analytical performance, and low cost [38]. At present, most of the light sources used by the PEC sensing platform are ultraviolet lamps or high-power xenon. These light sources are expensive and difficult to perform. Therefore, the PEC sensing platform of Cu-TCPP@MOFs/GCE adopts a low-cost visible light source as the excitation light source, which effectively reduces the practical application cost of the PEC sensing platform.

#### 3.7.1. Wavelength Selection

**Figure 8:**
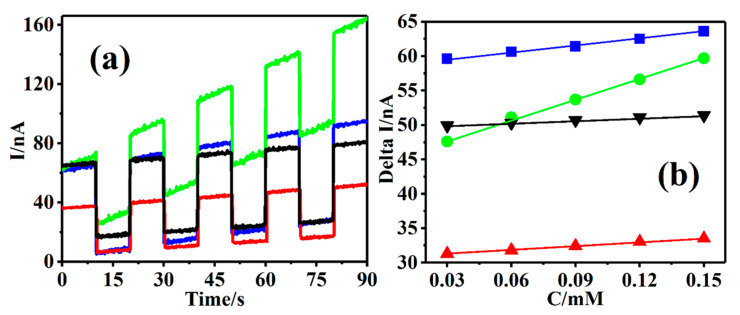
(**a**) Photocurrent responses of Cu-TCPP@MOFs/GCE in different concentrations of AA solution for the addition of 0.015 mM AA each time added 10 μL in 0.1 M PBS (pH = 7.0) at a 10 s regular time interval under blue light (blue curve), green light (green cure), red light (red curve), and white light (black curve). (**b**) The standard curves of the delta current varying with different concentrations of AA under four light conditions, blue light (blue line), green light (green line), red light (red line), and white light (black line), respectively.

The Cu-TCPP@MOFs sensing platform as a PEC sensor quantitatively detected AA based on the strong absorption properties of Cu-TCPP SBUs in Cu-TCPP@MOFs in visible light region of 400~700 nm. Figure 8a shows the photocurrent responses of Cu-TCPP@MOFs/GCE under AA concentration, ranging from 30 µM to 150 µM and the different wavelength light sources with “on-off-on” with every 10 s, including blue light with an effective wavelength of 420~470 nm, green light with an effective wavelength of 520~545 nm, red light with an effective wavelength of 620~630 nm, and white light with an effective wavelength of 400~700 nm. It is seen that Cu-TCPP@MOFs/GCE shows superior photocurrent responses under four light irradiation conditions, which confirms the excellent photocatalytic activities in visible light regions. However, in the four different wavelength irradiations, the photocurrent response of Cu-TCPP@MOFs/GCE under blue and green light irradiation was relatively higher than that under white and red-light irradiation, which could be attributed to the strong absorption at 424 nm and 539 nm of Cu-TCPP@MOFs. Moreover, the changes in the photocurrent responses at Cu-TCPP@MOFs/GCE, with AA concentration varying from 30 µM to 150 µM under the above-mentioned four light irradiations (Figure 8b), the corresponding linear equations are I(nA) = 49.54 + 0.013C(μM), R^2^ = 0.997(white light); I(nA) = 44.83 + 0.099C(μM), R^2^ = 0.998 (green light); I(nA) = 58.57 + 0.033C(μM), R^2^ = 0.997 (blue light); I(nA) = 30.73 + 0.019C(μM); and R^2^ = 0.998 (red light). The slope of the corresponding linear equations represented the sensitivity of the electrochemical sensor to the detected targeted molecule. The order of the slope is 0.099 of green light > 0.033 of blue light > 0.019 of red light > 0.013 of white light. Therefore, the green light was selected as the ideal excitation light source of the PEC sensing platform of Cu-TCPP@MOFs/GCE to detect AA in the present constructed Cu-TCPP@MOFs/GCE.

#### 3.7.2. Construction of Cu-TCPP@MOFs/GCE “On-Off-On” PEC Sensing Platform 

**Figure 9:**
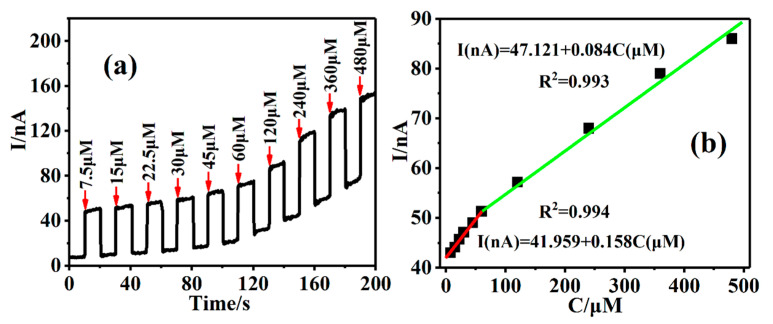
(**a**) Amperometric I-t curve of Cu-TCPP@MOFs/GCE in different concentrations of AA solution in 0.1 M PBS (pH = 7) at a regular time interval of 10 s (applied potential was 0.1 V) by PEC detection. (**b**) The standard curves of the peak currents versus different concentrations of AA solution.

Furthermore, the construction of Cu-TCPP@MOFs/GCE as a PEC sensing platform was used to detect AA based on the above irradiation wavelength optimization selection results. The photocurrent response intensity of PEC based on Cu-TCPP@MOFs/GCE enhanced with the increase in AA concentrations in 0.1 M PBS (pH = 7.0) solution under green light irritations, which was shown in Figure 9a. After adding AA concentration ranges from 7.5 μM to 480 μM, the photocurrent response of Cu-TCPP@MOFs/GCE increased from 50.1 nA to 146.5 nA, which provides a good theoretical basis and experimental model for the constructed PEC sensing platform to detect AA in visible light regions. Furthermore, the corresponding linear regression equation is established under different AA concentration ranges (Figure 9b). When AA concentration ranges from 7.5 μM to 60 μM, the corresponding linear equation is I(nA) = 41.959 + 0.158C(μM) R^2^ = 0.994 with LOD of 0.023 μM (S/N = 3) for AA, the sensitivity of 0.158 nA/μM; AA concentration ranges from 60 μM to 480 μM, the corresponding linear equation is I(nA) = 47.121 + 0.084C(μM) R^2^ = 0.993 with LOD of 0.043 μM (S/N = 3) for AA, the sensitivity of 0.084 nA/μM. 

### 3.8. Reproducibility, Stability, and Selectivity of Cu-TCPP@MOFs/GCE

**Figure 10:**
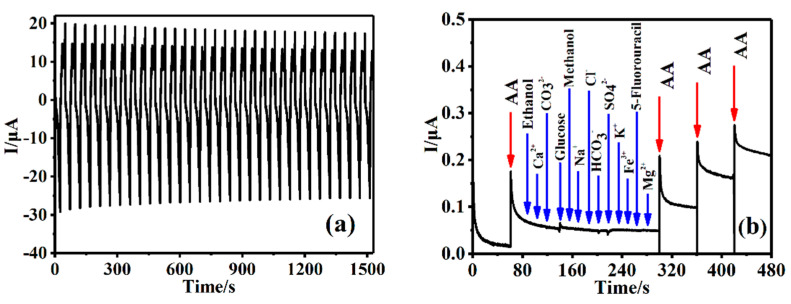
(**a**) CV of Cu-TCPP@MOFs/GCE in 0.3 mM AA solution for 32 cycles (n = 32). (**b**) Amperometric I-t curve of Cu-TCPP@MOFs/GCE for addition of AA and each 500 μM of potentially interferents, in 0.1 M PBS (pH = 7.0) (applied potential was 0.1 V).

To evaluate the application capability of the prepared sensor, the reproducibility, stability, and selectivity of Cu-TCPP@MOFs/GCE were investigated. For CV of Cu-TCPP@MOFs/GCE scanning in PBS for 32 cycles, the relative standard deviation (RSD) is 1.76%; this has been reported in our published paper [33]. Figure 10a shows CV of Cu-TCPP@MOFs/GCE scanning in 0.3 mM AA solution for 32 cycles, the relative standard deviation (RSD) is 1.71%. These results indicate that Cu-TCPP@MOFs/GCE demonstrates an outstanding detecting reproducibility in PBS and AA solution, and Cu-TCPP@MOFs/GCE for AA detection has acceptable repeatability.

The long-term stability of Cu-TCPP@MOFs/GCE was evaluated by measuring the CV of AA solution at three different concentrations (40, 80, and 120 μM) in 0.1 M PBS (pH = 7.0) solution every 7 days. The peak currents of 40, 80, and 120 μM AA remains 94.4%, 93.5%, and 93.1% of the initial values after 4 weeks of storage, respectively, which indicates that Cu-TCPP@MOFs/GCE demonstrates outstanding long-termed stability. To evaluate the selectivity and anti-interference of Cu-TCPP@MOFs/GCE, the influence of some possible common inorganic ions and organic compounds on the determination of AA was investigated by using the Amperometry I-t curve method (Figure 10b). By increasing AA concentration step by step, the current intensity gradually increased with the increase in AA concentration, and then some possible potential interferences (500 μM) were added, such as Mg^2+^, Ca^2+^, Na^+^, K^+^, Fe^3+^, SO_4_^2−^, HCO_3_^−^, CO_3_^2−^, Cl^−^, 5-Fluorouracil, Methanol, Ethanol and glucose. The results show that the current intensity of Cu-TCPP@MOFs/GCE hardly changed with the increase in the possible potential interferences of the measured solution. After adding all the interfering substances and continuing to increase AA concentration, the peak current intensity continued to display a ladder-shaped increase with the increase in AA concentration, which indicated that the peak current intensity was only related to AA concentration and had nothing to do with other distractions. Therefore, Cu-TCPP@MOFs/GCE demonstrates highly selective and anti-interference abilities for the determination of AA.

In order to further understand the advantages of the present sensor based on Cu-TCPP@MOFs/GCE, the performances of the previously reported sensors constructed by different materials were compared (Table 1). The results show the Cu-TCPP@MOFs/GCE sensor possesses good long-term stability, excellent reproducibility and selectivity, lower detection limit, and higher sensitivity relative to other reported sensors, which have better application potential in practice. The present sensor based on Cu-TCPP@MOFs could be applied to many different electrochemical technologies and offer a perfect linear response for AA, an ideal lower detection limit and a wide AA concentration detection range, compared with some previous reports. Furthermore, the current sensor based on Cu-TCPP@MOFs could be used as a PEC sensing platform because the Cu-TCPP ligand of Cu-TCPP@MOFs has strong absorption in the visible region, which could further improve the lowest detection limitation of target molecules.

### 3.9. The Mechanism of Oxidation Processes of AA

AA was the most common electroactive biological compound, being easily oxidized, and this constitutes the basis of its electrochemical determination. AA has a *γ*-lactone structure with two dissociable protons (pKa 4.04 and 11.34) [52]. The oxidation of AA involves the release of two electrons and two protons. Dehydroascorbic acid (DHA) was the product expected when two H atoms were abstracted from AA in a typical two-equivalent oxidation at a neutral pH [53]. 

**Scheme 1:**
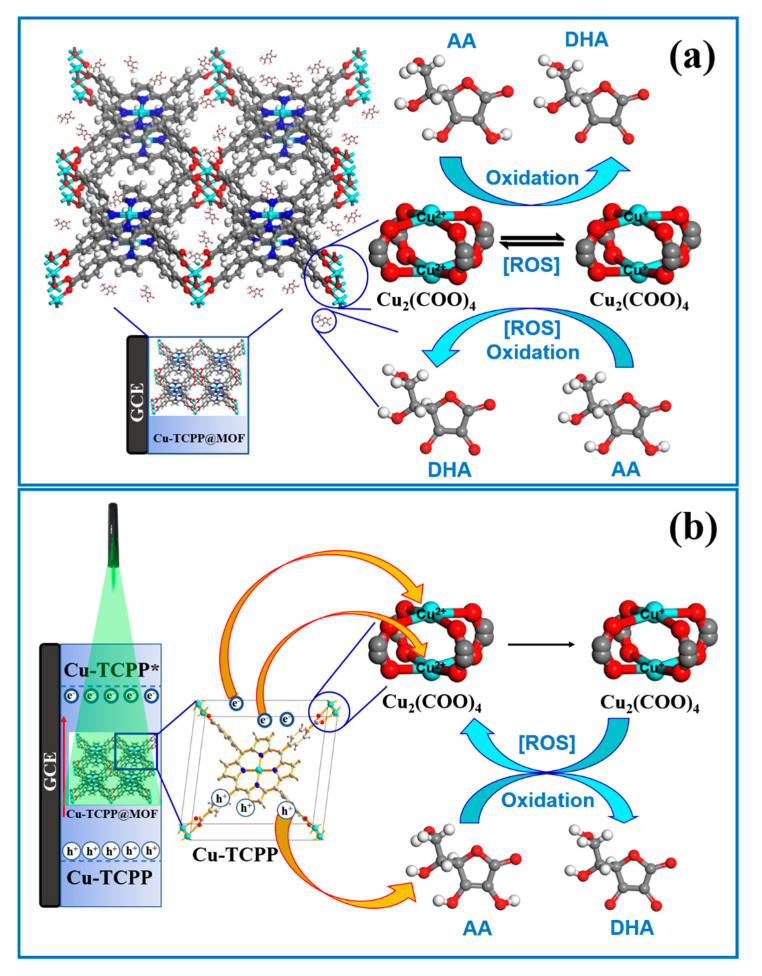
(**a**) The mechanism of Cu-TCPP@MOFs/GCE electrochemical sensing platform detected AA. (**b**) The mechanism of Cu-TCPP@MOFs/GCE PEC sensing platform detected AA.

Based on the above experimental data, the possible electrochemical sensing mechanism of Cu-TCPP@MOFs/GCE was proposed and shown in Scheme 1a. Firstly, AA is absorbed onto Cu-TCPP@MOFs and the Cu^2+^ of Cu_2_(COO)_4_ in Cu-TCPP@MOFs are easily activated by AA molecule, which resulted in reduced Cu^2+^ to Cu^+^ under the external electric field [54] and a formed Cu^+^ transition intermediate state in Cu-TCPP@MOFs [55]. The strong oxidation activities of Cu^+^ catalyzes the AA oxidation process and makes AA molecule oxidize into DHA. The generated Cu^+^ further reduces O_2_ to generate a series of distinct reactive oxygen species (ROSs). Meanwhile, Cu^+^ is oxidized back to Cu^2+^ [61].

Scheme 1b illustrates the possible sensing mechanism of Cu-TCPP@MOFs/GCE as a PEC sensor under green light irradiations [33]. Cu-TCPP is an ingredient in Cu-TCPP@MOFs and acts as a complementary antenna absorbed and exited by light energy. The photogenerated electrons and photogenerated holes are effectively separated in the excited states. The photogenerated electrons were transferred to Cu_2_(COO)_4_ ingredient in Cu-TCPP@MOFs via intramolecular electron transfer and Cu^2+^ of Cu_2_(COO)_4_ is reduced to Cu^+^. The strong oxidation activity of Cu^+^ could effectively promote the catalytic oxidation abilities of Cu-TCPP@MOFs towards AA [54] and make AA molecules oxidate into DHA. The generated Cu^+^ further reduces O_2_ to generate a series of distinct reactive oxygen species (ROSs). Meanwhile, Cu^+^ is oxidized back to Cu^2+^ [61]. In addition, the photogenerated holes will directly oxidize AA molecules into DHA, which would greatly reduce the recombination ratios of photogenerated holes and photogenerated electrons. Therefore, the addition of AA molecules could effectively increase the photocurrent response intensity, which results that the sensitivity and LOD of the PEC sensor based on Cu-TCPP@MOFs being superior to other electrochemical methods [58].

### 3.10. Analytical Applications

#### 3.10.1. The Accuracy of Cu-TCPP@MOFs/GCE Detection Platform

The accuracy of the Cu-TCPP@MOFs/GCE detection platform was verified by an AA test standard kit. Cu-TCPP@MOFs/GCE detection platform was confirmed by comparing the results of the AA test standard kit to simultaneously detect the content of AA in the same actual serum samples. Before CV, DPV, CA, and PEC detection, four human serum samples were diluted with 0.1 M PBS by 800 times, respectively. The absorbance of tested samples was calculated according to equation C_1_ = 500 μmol/L (A_1_/A_0_). C_1_ was the concentration of the sample. A_1_ and A_0_ were absorbance of sample and standard substance, respectively. The test values of real samples by the two test methods were listed in Table 2. The results displayed that the test data of Cu-TCPP@MOFs/GCE detection platform are almost consistent with that of the standard kit and the error value is less than ± 2%. Therefore, the accuracy of Cu-TCPP@MOFs/GCE detection platform fully satisfies the requirements of AA detection standards in the practical testing samples.

#### 3.10.2. The Applicability of Cu-TCPP@MOFs/GCE Detection Platform

To further evaluate the analytical applicability of the Cu-TCPP@MOFs/GCE sensing platform, Cu-TCPP@MOFs/GCE performed the determination of AA in human serum samples. Before CV detection, two human serum samples were diluted with 0.1 M PBS 400 times, respectively. Additionally, the applicability of the PEC sensing platform of Cu-TCPP@MOFs/GCE was further tested for AA analysis in human serum. Before PEC detection, the other two human serum samples were filtered and diluted with 0.1 M PBS 500 times. The PEC sensing platform of Cu-TCPP@MOFs/GCE, using Cu-TCPP@MOFs as the electrode, achieved sensitive and selective determination of AA in the real sample. The results of the real sample analysis were listed in Table 3. The relative standard deviations (RSD) are in the range of 0.42~1.18%, with the spiked recoveries in the range of 94.8~102.2%. They represented remarkable practicality for the detection of AA in real human serum samples. The results show that Cu-TCPP@MOFs sensing platform achieved sensitive and selective determination of AA in the real sample. 

## 4. Conclusions

In this work, a sensitive, facile, rapid, and cost-effective electrochemical and PEC sensor based on Cu-TCPP@MOFs thin films was designed and fabricated, and successfully applied to detect AA. Thanks to Cu-TCPP@MOFs thin films with a larger specific surface area, more exposed active centers, and smaller diffusion resistance relative to that of Cu-TCPP@MOFs bulk materials, Cu-TCPP@MOFs thin films have superior electrochemical and PEC activities. The well linear regression equations were established between the peak current intensity and AA concentrations in the different electrochemical technologies including CV, DPV, and CA. AA concentration ranges applicable to various electrochemical equations were as follows: 0.45~2.10 mM of CV, 0.75~2.025 mM of DPV, 0.3~2.4 mM of CA, and the corresponding detection limits for AA were 1.08 μM (S/N = 3), 0.14 μM (S/N = 3) and 0.049 μM (S/N = 3), respectively. Moreover, Cu-TCPP@MOFs were used as a PEC sensing platform to quantitatively detect AA. The photocurrent response of the PEC sensing platform based on Cu-TCPP@MOFs/GCE towards AA exhibits a wide linear AA concentration range of 7.5~480 μM. When AA concentration ranges from 7.5 μM to 60 μM, the corresponding linear equation is I(nA) = 41.959 + 0.158C(μM), R^2^ = 0.994 with LOD of 0.023 μM (S/N = 3) for AA, the sensitivity of 0.158 nA/μM; AA concentration ranges from 60 μM to 480 μM, the corresponding linear equation is I(nA) = 47.121 + 0.084C(μM), R^2^ = 0.993 with LOD of 0.043 μM (S/N = 3) for AA, the sensitivity of 0.084 nA/μM.

## Figures and Tables

**Table 1:** Comparison of analytical figure of merits for electrochemical detection of AA using different types of modified electrodes.

Electrode	Electrolyte (pH)	Technique	Linear Working Range (µM)	Detection Limit (µM)	Ref.
Poly(EBT)/GCE	0.05 M PBS (pH 4.0)	DPV	150–1000	10	[39]
GCE/ZnO/RM	0.10 M PBS (pH 7.0)	CV	15–240	1.4	[40]
Pd-CNFs/CPE	0.10 M PBS (pH 4.5)	DPV	50–4000	15	[41]
Chitosan-Graphene/GCE	0.05 M PBS (pH 7.0)	DPV	50–1200	50	[42]
Nano-Au/p-TA/GCE	0.10 M PBS (pH 4.0)	DPV	2.1–50.1	1.1	[43]
SGNF/IL/CS/GCE	0.10 M PBS (pH 6.0)	DPV	0.12–260	14.8	[44]
PCFE	0.10 M PBS (pH 4.0)	DPV	10.0–567.1	2.9	[45]
SPGNE	0.10 M PBS (pH 7.0)	DPV	4.0–4500	0.95	[46]
AuNPs-β-CD-Gra/GCE	0.10 M NaH_2_PO_4_-HCl (pH 2.0)	SWV	30–2000	10	[47]
NanoSnO_2_/MWCNTs/CPE	19.6 mL PBS (pH 6.4) containing 0.10 M KCl	DPV	0.1–5	50	[48]
(N-PCNPs)/GCE	0.10 M PBS (pH 7.0)	DPV	80–2000	0.74	[49]
Pd_3_Pt_1_/PDDA-RGO/GCE	0.10 M PBS (pH 7.4)	DPV	40–1200	0.61	[50]
P-4-ABA/GCE	0.20 M PBS (pH 4.5)	DPV	20–800	5.0	[51]
Poly(Tyr)/MWCNTs-COOH/GCE	0.067 M PBS (pH 7.4)	DPV	50–1000	2.0	[52]
Fe_3_O_4_/rGO/GCE	0.10 M PBS (pH 6.5)	DPV	1000–9000	0.42	[53]
CPE/MWCNTs/IL/PdNPs	0.10 M PBS (pH 5.0)	DPV	0.6–112	0.20	[54]
PImox-GO/GCE	0.10 M PBS (pH 3.0)	DPV	75–2275	18	[55]
PTCA/PDAox/GCE	0.10 M PBS (pH 3.0)	DPV	76–3900	25.3	[56]
AuNPs@MoS_2_/GCE	0.10 M PBS (pH 7.0)	DPV	50–100,000	100	[57]
BN/GCE	0.10 M PBS (pH 6.5)	DPV	30–1000	3.77	[58]
HNGA/GCE	0.10 M PBS (pH 7.4)	DPV	50–1500	16.7	[59]
Ti-C-T_x_/GCE	0.10 M PBS (pH 7.4)	DPV	100–1000	4.64	[60]
Cu-TCPP@MOFs/GCE	0.10 M PBS (pH 7.0)	CV, DPV, CA, and PEC	450–2100 (CV), 750–2025 (DPV), 300–2400 (CA), 7.5–480 (PEC)	1.08 (CV), 0.14 (DPV), 0.049 (CA), 0.023 (PEC)	This Work

**Table 2:** Results of the determination of AA in different samples (n = 5).

	Serum 1	Serum 2	Serum 3	Serum 4
Detection method of detection platform	CV	DPV	CA	PEC
Detection platform/mM	0.463 ± 0.010	0.379 ± 0.004	0.398 ± 0.006	0.367 ± 0.005
AA test standard kit/mM	0.456 ± 0.009	0.383 ± 0.007	0.393±0.008	0.368 ± 0.007
Error value/%	1.54	−0.10	1.27	−0.27

**Table 3:** Results of the determination of AA in different samples (n = 5).

	Serum 1	Serum 2		Serum 3	Serum 4
Detection method	CV	CV	Detection method	PEC	PEC
Detected/mM	0.957 ± 0.019	0.934 ± 0.018	Detected/μM	0.729 ± 0.014	0.650 ± 0.011
Added/mM	1.0	1.0	Added/μM	0.5	0.5
Found/mM	1.913 ± 0.030	1.905 ± 0.024	Found/μM	1.203 ± 0.016	1.161 ± 0.004
Total/mM	382.80	373.60	Total/μM	364.50	325.00
Recovery/%	95.60	97.10	Recovery/%	94.80	102.20
RSD/%	0.78	1.18	RSD/%	0.61	0.42

## Data Availability

The data that support the findings of this study are openly in http://doi.org/.
